# Effect of raw electroencephalogram-guided anesthesia administration on postoperative outcomes in elderly patients undergoing abdominal major surgery: a randomized controlled trial

**DOI:** 10.1186/s12871-023-02297-5

**Published:** 2023-10-06

**Authors:** Ziqing He, Hao Zhang, Yahui Xing, Jia Liu, Yang Gao, Erwei Gu, Lei Zhang, Lijian Chen

**Affiliations:** Department of Anesthesiology, The First Affiliated Hospital of Anhui Medical University, No.218 Jixi Road, Hefei, Anhui Province 230022 China

**Keywords:** Anesthesia depth, Raw electroencephalogram, Elderly, Frontal alpha, Vulnerable patients, Postoperative outcomes

## Abstract

**Background:**

EEG monitoring techniques are receiving increasing clinical attention as a common method of reflecting the depth of sedation in the perioperative period. The influence of depth of sedation indices such as the bispectral index (BIS) generated by the processed electroencephalogram (pEEG) machine to guide the management of anesthetic depth of sedation on postoperative outcome remains controversial. This research was designed to decide whether an anesthetic agent exposure determined by raw electroencephalogram (rEEG) can influence anesthetic management and cause different EEG patterns and affect various patient outcomes.

**Methods:**

A total of 141 participants aged ≥ 60 years undergoing abdominal major surgery were randomized to rEEG-guided anesthesia or routine care group. The rEEG-guided anesthesia group had propofol titrated to keep the rEEG waveform at the C-D sedation depth during surgery, while in the routine care group the anesthetist was masked to the patient’s rEEG waveform and guided the anesthetic management only through clinical experience. The primary outcome was the presence of postoperative complications, the secondary outcomes included intraoperative anesthetic management and different EEG patterns.

**Results:**

There were no statistically significant differences in the occurrence of postoperative respiratory, circulatory, neurological and gastrointestinal complications. Further EEG analysis revealed that lower frontal alpha power was significantly associated with a higher incidence of POD, and that rEEG-guidance not only reduced the duration of deeper anesthesia in patients with lower frontal alpha power, but also allowed patients with higher frontal alpha power to receive deeper and more appropriate depths of anesthesia than in the routine care group.

**Conclusions:**

In elderly patients undergoing major abdominal surgery, rEEG-guided anesthesia did not reduce the incidence of postoperative respiratory, circulatory, neurological and gastrointestinal complications. rEEG-guided anesthesia management reduced the duration of intraoperative BS in patients and the duration of over-deep sedation in patients with lower frontal alpha waves under anesthesia, and there was a strong association between lower frontal alpha power under anesthesia and the development of POD. rEEG-guided anesthesia may improve the prognosis of patients with vulnerable brains by improving the early identification of frail elderly patients and providing them with a more effective individualized anesthetic managements.

## Introduction

Intraoperative neuromonitoring allows monitoring of the changes in brain electrical activity during the changing states of consciousness under general anesthesia and offers information on anesthesia depth. EEG monitoring can help anesthesiologists to avoid the use of unnecessary high anesthetics doses, which is able to be a risk factor for occurring peri-operative neurocognitive disorders [1]. In the observational studies, it is pointed out that excessive exposure to potent volatile agents is likely to enhance the incidence of postoperative delirium (POD), which is linked to adverse outcomes and increased resource utilization [2]. At present, the bispectral index (BIS) serves as the most generally employed processed electroencephalogram (pEEG) device for the reason of research and clinics [3]. A number of studies [4, 5] have found that BIS guidance would result in less anesthetic exposure and therefore “lighter” anesthesia, thereby decreasing the risk of POD compared with deeper general anesthesia. However, in a study by Short et al [6] in which 6500 high-risk elderly patients were randomized to either a deeper (BIS 35) or lighter (BIS 50) sedation group, no significant differences were found in one-year all-cause mortality and other clinical outcomes. A study by Wildes et al [7] also showes no significant effect of BIS monitoring on the occurrence of POD. There are a number of possible reasons for the inconsistent results of the relevant studies, including the limitations of pEEG monitoring as represented by the BIS in clinical use. The pEEG, which defines an equivalent anesthetic state independent of anesthetic medication and patient age by the same value, is based on an algorithm that simplifies information about anesthetic sedation and does not identify the underlying EEG features associated with the patient’s state [8], compromising the reliability of the pEEG index as an EEG depth indicator and prompting researchers to explore more accurate methods of managing the depth of anesthetic sedation.

The raw electroencephalogram (rEEG) correlates significantly with the level of consciousness in patients under general anesthesia [9], and the EEG pattern varies according to the depth of anesthesia and sedation [10]. According to the revised version of Kugler’s EEG analysis method [11](Table 1), the depth of anesthesia can be refined into six levels, A, B, C, D, E and F, by analyzing the EEG characteristics at different depths of anesthesia [11]. The anesthetist can monitor and visually analyze the EEG during surgery to manage the depth of sedation and keep the patient in a C-D level of sedation by adjusting the dose or infusion rate of general anesthetic to avoid sedation that is too deep or too light. In certain research, it is proposed that the anesthetic dose and the presence or absence of certain electroencephalogram (EEG) patterns, including burst suppression (BS), are linked to the risk of subsequent cognitive disorders [7, 12]. In research settings, the intraoperative BS and alpha (8–12 Hz) oscillatory activity within the frontal EEG have been associated with POD [13–15] and preoperative cognitive impairment [16], respectively. In terms of adults, the size of the anesthesia-induced frontal alpha activity is linked to the age [17], cognitive status [16], and antinociception [18] as well as the development of EEG BS activity [14]. Taking into account the fact that numerous changes are observed in the brain anatomy and physiology linked to typical aging [11, 17], there are certain variations of EEG patterns of elderly patients and young patients under general anesthesia, and elderly patients may exhibit less alpha oscillatory activities under general anesthesia [19, 20] and tend to present with BS [21]. At the same time, anesthetic drugs act at sites within the brain that undergo profound changes during typical aging, in which a lot of insults are enhanced by the aging process and within the developing brain [22]. As a result, compared with that of younger patients, different methods are needed for the anesthetic management of older patients.

Given that the pEEG method of monitoring depth of sedation has been widely used in clinical practice, and that the limitations of the pEEG itself and the neurological changes in elderly patients lead to limitations in the accuracy of pEEG-guided anesthesia in elderly patients, the aim of this study was to investigate whether rEEG-based intraoperative visual analysis of EEG-guided anesthetic depth management could have an impact on intraoperative anesthetic management and EEG patterns and affect postoperative outcomes in elderly patients undergoing major abdominal surgery compared with routine anesthetic care monitoring, and to provide a reference for the clinical application of rEEG in elderly patients.

**Table 1:** Six levels of anesthesia

Stage	Frequency admixture/dominance in EEG per 30s epoch	Depth of anesthesia
A	α(8-12 Hz) and β (13-30 Hz) activity, with intermixed eye movement/blinking and myogenic artifact from talking/swallowing	Not applicable (awake)
B	Fast β and θ (4-7 Hz) but rare δ (1-3 Hz)	Light
C	δ activity for at least 20% but no more than 50% of epoch	Light to moderate
D	δ activity for at least 50% of epoch; brief periods of suppression not to exceed 10 s	Moderate
E	Burst-suppression pattern, with at least 10 s but no more than 20 s of suppression per epoch	Profound
F	Burst-suppression pattern, with at least 20 s of suppression per epoch	Very profound

## Materials and methods

This single-center and randomized controlled trial compared the outcomes of two parallel groups, which were the rEEG-guided group and the routine care group. The research was approved by the Clinical Research Ethics Committee of the First Affiliated Hospital of Anhui Medical University Institutional Review Board (IRB number: No. PJ2020-13-09), and written informed consent was obtained from each subject that attends the trial. In addition, the trial was registered before patient enrollment at the Chinese Clinical Trial Registry at 12/11/2020 (Clinical Trials Number: ChiCTR2000039864) and was conducted from November 2020 to May 2022 in accordance with the Helsinki Declaration.

### Participants

The male or female patients over 60 years old scheduled for elective major abdominal surgery were involved. Below present the exclusion criteria: patient refusal, a history of dementia or psychiatric illness, difficulty with follow-up, or poor compliance.

### Randomization, blinding, and allocation concealment

By using randomized closed envelopes, the randomization procedure was carried out by the responsible senior consultant. The group allocation was performed following the recruitment through the opening of the sequential envelopes. The anesthesiologists were not masked due to the nature of the anesthetic technique. The patients and research staff in charge of the postoperative patient assessments did not learn about the group assignment.

### Intervention

**Fig. 1:**
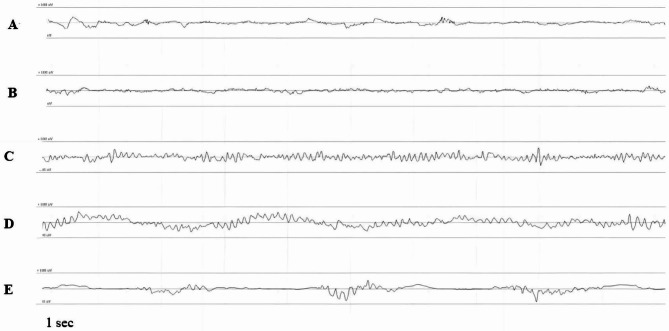
EEG tracing at various stages of anesthesia

After the participants entered the operation room, the five-lead electrocardiogram (ECG), invasive blood pressure via a radial artery, and pulse oxygen saturation (SpO_2_) were all monitored. Masimo Quatro sensors (Masimo, Irvine, CA, USA) were attached to the foreheads of all subjects. General anesthesia was induced with midazolam (0.02 mg/kg), etomidate (0.2 mg/kg), sufentanil (0.5 µg/kg), and cisatracurium (0.2 mg/kg), and kept with 0.1–0.3 µg/kg.min remifentanil when intubation ends. Both groups did not receive any volatile drugs. Intraoperatively, the propofol infusing rate was titrated to maintain the depth of sedation at a C-D level, spectrogram maintains constant slow wave/δ oscillation in the rEEG-guided group, if depth of anesthesia is switched from C-D, adjust propofol dose by 0.5 mg/kg body weight. The rEEG trace offers the primary guidance (Fig. 1). The depth of anesthesia was adjusted according to experience in the routine care group to maintain stable vital signs. In both groups, vasoactive drugs were used as needed to maintain MAP fluctuations within ± 20% [6, 23] of the baseline of the patient. Postoperative pain management was achieved by a patient-controlled intravenous analgesia pump (PCIA, 150 ml) that includes flurbiprofen 1.5-2 mg/kg + sufentanil 3.5–4.5 µg/kg. The locking time was 15 min, the background infusion rate was 2 ml/h, the controlled dose was 2 ml. Moreover, a 50 mg flurbiprofen intravenous injection was supplied as an analgesic rescue when the ward requires it.

### Outcomes and data collection

The demographic and hospital characteristics of the patients, such as age, body mass index, gender, coexisting medical conditions, type of operation (laparotomy or laparoscopy) were all recorded. Intraoperative data on the procedure and anesthesia were recorded, including time of induction of anesthesia (midazolam start time), time of start of operation (skin cut time), MAP and heart rate at all key intraoperative time points (baseline, at loss of consciousness, skin cut, every 30 min during the operation, at end of operation), time of end of operation (time of last suture) and time of end of anesthesia (propofol stop time). Intraoperative dose of maintenance (propofol, remifentanil) and additional drugs (sufentanil, cisatracurium), record of vasoactive drug use, time of patient admission to PACU and time of discharge from PACU. Patients were assessed for the occurrence of major organ system complications during hospitalization, postoperative length of stay and 30-day postoperative all-cause mortality. In the present study, the primary outcomes of interest were systemic complications within the hospitalization after surgery. Systemic complications were divided into respiratory complications [24] (pulmonary infection, pleural effusion, respiratory insufficiency, and atelectasis), cardiovascular complications [25, 26](i.e., hemodynamic instability, low cardiac output, new arrhythmias, ischemic heart disease, and cardiac dysfunction), neurological complications [24] (transient ischemic attack and delirium), gastrointestinal complications [27](nausea and vomiting), numerical rating scale (NRS) pain score of >3, and surgical site infection [24]. Based on the imaging examination, laboratory examination, and clinical symptoms, the incidence of postoperative complications was evaluated. The secondary outcomes included the incidence of BS and intraoperative management (i.e., anesthetic doses, electroencephalographic data, hemodynamics data). Suppression periods referred to periods which are above 0.5 s with voltage not exceeding nearly ± 5 µV [28]. For each electrode, alpha power was estimated through the computing of the average power between 8 and 12 Hz [29]. Each person that analyses EEG data was blinded to the group allocation.

### EEG processing

A 4-channel Sedline brain function monitor (Masimo, Irvine, CA, USA) was used for forehead EEG acquisition. The electrodes for the sensors record EEG between Fp1, Fp2, F7 and F8 with the ground electrode at Fpz, and the reference electrode at roughly 1 cm more than Fpz. A sampling rate of 178 Hz (16 bits) and a preamplifier bandwidth of 0.5–92 Hz was used to record the EEG data. In order to check the exact time and anesthesia level, an experienced researcher manually browsed EEG data of all of the patients. For the purpose of carrying out the spectral analysis, 10 s [30] of intraoperative, artifact-free and non-BS EEG were adopted. The spectrogram was computed by means of the multitape method which was realized within the MATLAB Chronux toolbox [31]. Below are the spectral analysis parameters: number of tapers, *K* = 5; window length T = 2 s with a 1.95 s overlap; spectral resolution = 3 Hz; and time-bandwidth product of TW = 3.

### Statistical analysis

In this trial, the sample size was estimated for an α level of 0.05 and 90% power in order to detect a 10% difference in the occurrence between groups. On the basis of the preliminary results, the incidence of adverse events after surgery was indicated to be 50% in the routine care group, and we calculated that a sample of 55 patients would provide 90% power to reduce it to 25% [7] in the rEEG-guided group after randomization with a two-sided α level of 0.05. This research aimed to recruit a total of 120 participants to account for missing data. The baseline characteristics were summed up by group through the use of medians and interquartile ranges (IQR), means and standard deviations (SD), or counts and percentages as appropriate. The reason to withdraw from this research at different stages and the participant’s disposition were recorded. Via the SPSS 25.0 software (SPSS, Inc., Chicago, IL, USA), statistical analyses were carried out. The Shapiro–Wilk test was employed to evaluate the continuous data normality. Quantitative data were in the form of mean ± SD or median (quartile distance) depending on their distribution. Normally distributed data were evaluated by using an independent two-sample t-test, whereas the other quantitative data were analyzed using the Mann–Whitney U test. For the purpose of comparing the categorical variables between the two groups, the Pearson *χ*^2^ test or Fisher’s exact test was employed. For measures that indicated a significant group by time interaction effects, the findings of the post hoc analysis on the differences between the two groups were assessed by means of an independent sample t-test with Bonferroni’s post-test correction. Considering all of the analyses, a *p* value of < 0.05 indicates a statistically significant difference. GraphPad Prism 8.0 (GraphPad Software, La Jolla, California, USA) was adopted in order to establish the figures.

## Results

**Fig. 2:**
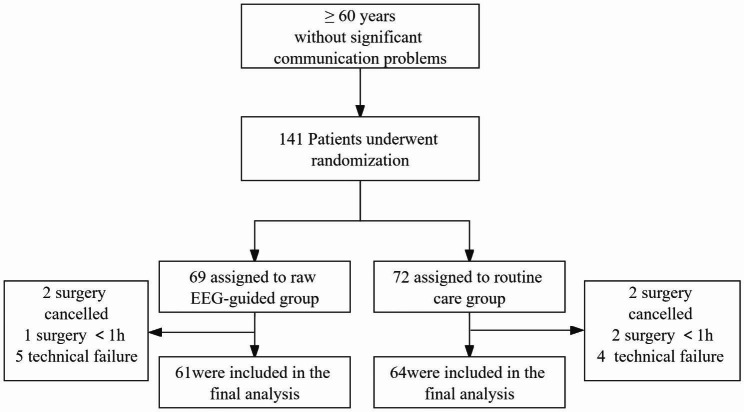
Flow diagram of included patients

141 patients that had undergone abdominal major surgeries from November 2020 to May 2022 (e.g., gastrointestinal, hepatobiliary-pancreatic) were included in the trial. Among them, 69 were randomized to the rEEG-guided group and 72 to the routine care group, respectively. Taking into account the 5 patients within the rEEG-guided group and 4 within the routine care group, a technical failure of EEG monitoring prevented the clinicians from analyzing the EEG results. Missing data was distributed almost evenly between the two groups, the final 125 patients were included in the outcome analysis, 61 in the rEEG-guided anesthesia management group and 64 in the conventional anesthesia management group (Fig. 2). Furthermore, no difference was observed in any of the measured baseline variables in both groups, including demographic characteristics, comorbid health conditions and preoperative evaluation of ASA stage (Table 2).

**Table 2:** Patient characteristics in each group

	rEEG-guided group	Routine care group	*P*
**Age, years**	68[65 73]	69[65 74]	0.203
**Sex, no.**			0.702
Male	39(64%)	43(67%)	
Female	22(36%)	21(33%)	
**Body mass index, kg/m2**	22[20 24]	22[20 25]	0.758
**ASA physical status,no.**			0.173
1	0	0	
2	42(69%)	36(56%)	
3	18(29%)	28(44%)	
4	1(2%)	0	
**Planned postoperative care in ICU,no.**	1(2%)	1(2%)	1.000
**Coexisting medical conditions,no.**			
Cardiovascular disease	32(52%)	43(67%)	0.093
Stroke or neurological disease	9(15%)	12(19%)	0.55
Respiratory disease	20(33%)	18(28.1%)	0.698
Diabetes	10(16%)	8(13%)	0.535
Rheumatoid arthritis or connective tissue disease	1(2%)	2(3%)	1.000
Renal disease	2(3%)	2(3%)	1.000
Liver disease	4(7%)	3(5%)	0.948

Values are presented as number (%) or median (1Q, 3Q). ASA: American Society of Anesthesiologists classification; ICU: intensive care unit

### Primary outcomes

There were no intraoperative complications in either group. There was no statistically significant difference in respiratory, cardiovascular, gastrointestinal and neurological complications between the two groups (P > 0.05). The incidence of POD was 3% in the rEEG-guided group compared to 11% in the routine care group, indicating no significant difference (P > 0.05). Postoperative pain was assessed using the NRS scale and no significant difference was found between the two groups (P > 0.05). In addition, the rEEG guidance showed no effect on length of hospital stay or postoperative hospital stay (P > 0.05) or all-cause mortality at 30 days postoperatively (P > 0.05) (Table 3).

**Table 3:** Postoperative Outcomes

	rEEG-guided group	Routine care group	*P*
**Cardiovascular complications,no.**	5(8%)	5(8%)	1.000
**Respiratory complications,no.**	3(5%)	6(9%)	0.537
**Cerebral infarction or hemorrhage,no.**	0	1(2%)	1.000
**POD**	2(3%)	7(11%)	0.190
**Gastrointestinal complications,no.**	21(34.4%)	30(46.9%)	0.151
**Surgical site infection,no.**	2(3%)	3(5%)	1.000
**NRS pain-score >3, no.**	24(39%)	29(45%)	0.500
**Intraoperative awareness,no.**	0	0	/
**Duration of hospital stay,d**	14[11 17]	15[12 19]	0.108
**Duration of postoperative hospital stay,d**	9[7 11]	10[8 14]	0.145
**Prolonged hospitalization**	6(10%)	13(20%)	0.103
**Mortality up to 30d after surgical procedure,no.**	1(2%)	0	0.488

Values are presented as number (%) or median (1Q, 3Q). ICU: intensive care unit; NRS: numerical rating scale; POD: postoperative delirium

### Secondary outcomes

In both groups, no statistically significant difference was found in the surgery type or location. There was a total of 5 patients with BS, with an incidence of 8% and a median BS duration of 5.3s in the rEEG-guided group, while the routine care group had 12 patients with BS. There was no statistically significant difference in the incidence of BS between the two groups (P > 0.05), while the duration of BS in the routine care group was longer than that in the rEEG-guided group (P < 0.05). The median duration of anesthesia was 155.0 (IQR 120.5–213.0) in the rEEG-guided group and 160.0 (128.5–190.5) in the routine care group, meaning no significant difference. Moreover, there were no significantly different doses of propofol, opioids and neuromuscular blocking agents in both groups. During the maintenance of anesthesia, MAP was 90 mmHg within the rEEG-guided group and 91 mmHg within the routine care group, therefore showing no significant difference, and there was no statistically significant difference between basal and intraoperative MAP at any time point in either group. Furthermore, no inversely significant difference was observed in the time taken in PACU between the two groups (Table 4).

**Table 4:** Perioperative Care Measures

	rEEG-guided group	Routine care group	*P*
**Location of surgery**			0.954
upper abdominal	34(56%)	36(56%)	
down abdominal	27(44%)	28(44%)	
**Type of surgery**			0.746
Open	37(61%)	37(58%)	
Laparoscopic	24(39%)	27(42%)	
**Duration of anesthesia, min**	155.0 [120.5 213.0]	160 0.0[128.5 190.5]	0.843
**Additional drugs**			
Propofol,mg	640.5 [452.5 1000.0]	637.0 [520.0 847.5]	0.998
Remifentanil,µg	1250.0 [906 1850]	1373.0 [1081 1788]	0.224
Sufentanil ,µg	20.0[16.0 25.0]	20.0 [20.0 25.0]	0.53
Cisatracurium ,mg	10.0[6.0 13.0]	10.0 [6.0 13.0]	0.727
**Any vasoconstrictor, no**	23(38%)	18(28%)	0.254
**BS, no.**	5(8%)	12(18%)	0.085
**Duration of BS (s)**	5.3 [0, 31.5]	25.0 [3.4, 180.5]	0.018
**Intraoperative movements**	0	0	/
Admitted to PACU from OR, no.	61	64	/
Time spent in the PACU, min	42.5 [35.0 50.0]	45.0 [36.0 55.0]	0.083

Values are presented as number (%), mean ± SD or median (1Q, 3Q). MAP: mean arterial pressure; BS: burst suppression; PACU: post-anesthesia care unit

### Subgroup analyses

#### Frontal alpha power and POD

**Fig. 3:**
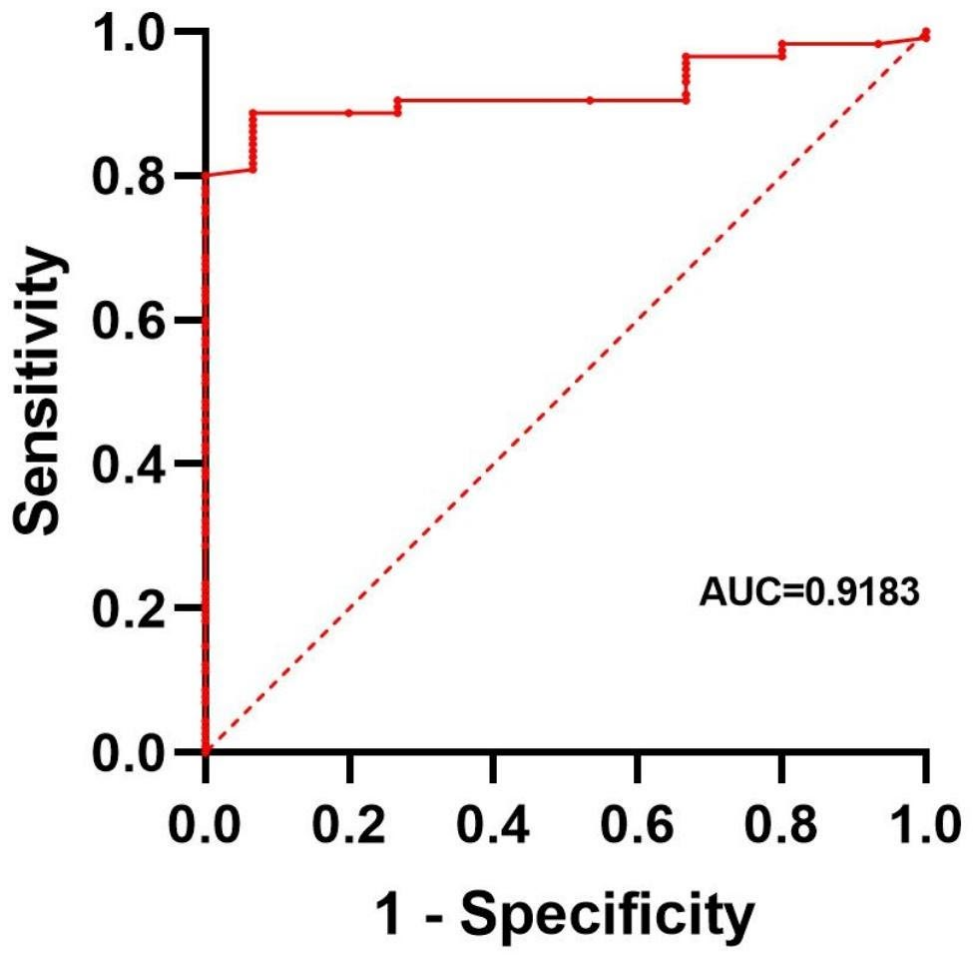
The receiving operating characteristic curves for the model

Patients that suffer from a lower alpha power are reported to be more prone to the development of BS under anesthesia [32]. Taking into account the relationship between intraoperative BS and POD [14], this research characterized the connection between POD and a series of possible predicting factors, i.e., age, sex, anesthesia-induced frontal alpha power and the rate of intraoperative propofol boluses [32], as calculated through the division of the cumulative dose of propofol by the operation time interval via using the logistic regression analysis. *p* values of < 0.05 were determined to be a significant relationship between per variable and the result. At last, within the full model, just the alpha power (estimated OR = 0.763 through referring to a 1 dB increase in the alpha power, with a 95% confidence interval [CI], 0.655–0.888, *p* < 0.001) still had a significant linkage to POD probability, whatever the group is. In order to show the comparable AUC performance, area below the model’s receiver operating characteristic curve was counted (Fig. 3).

#### Subgroup analysis on sedation and postoperative complications

**Fig. 4:**
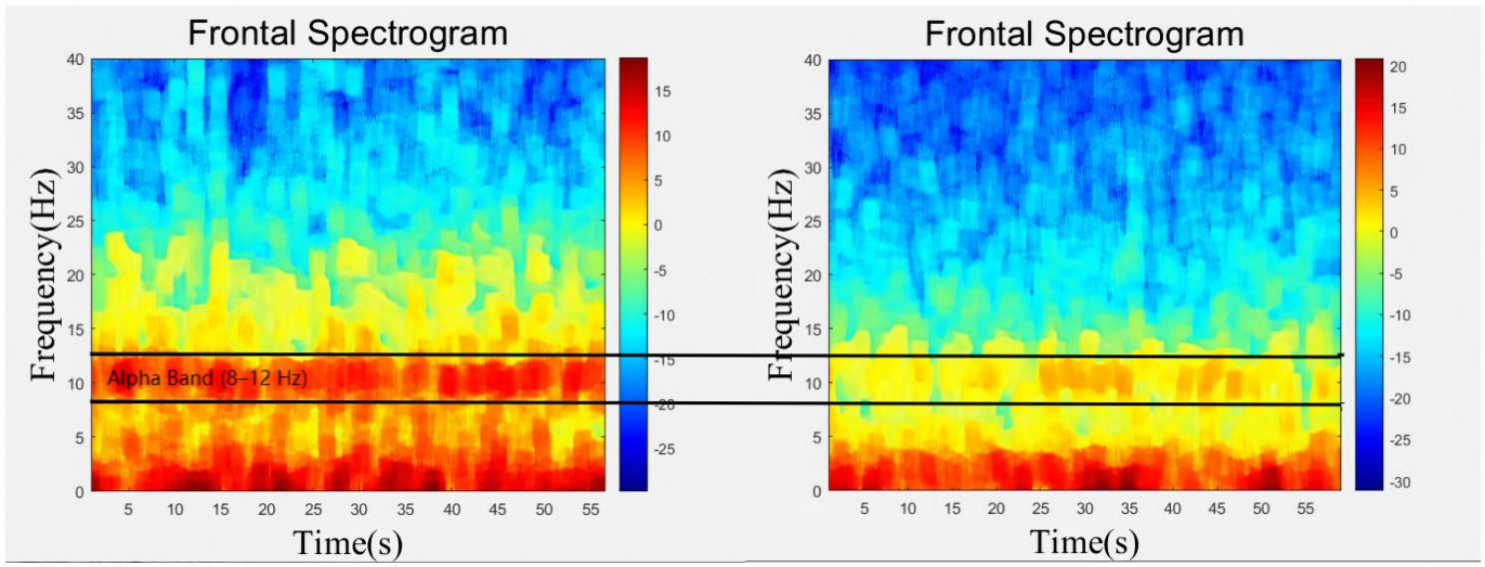
Examples of high(left) and low(right) alpha power within a left frontal spectral display. The vertical axis is frequency (Hz). The blue and red colors represent low and high power (dB). The horizontal axis is time (s). The dark horizontal lines present the alpha band range (8–12 Hz)

**Fig. 5:**
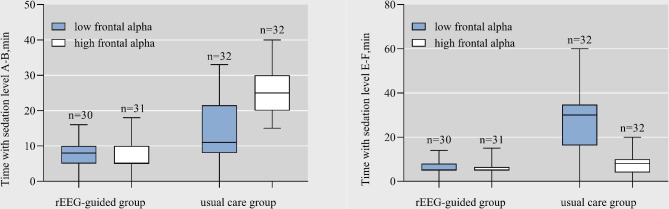
The box-and-whisker plots show the medians (thick horizontal lines) and interquartile ranges (IQRs; boundaries of the box) and ranges. Whisker boundaries are set at 1.5 × IQR. The sedation time plots depict the cumulative times in each of the study groups during which the electroencephalogram sedation ratio was > 1%

Alpha oscillations can be viewed to be a neurophysiological biomarker of brain vulnerability [16]^,^ [11] and are likely to assist in finding out patients that suffer from a higher risk of postoperative neurocognitive impairment and lower anesthetic requirements. As a result, a subgroup analysis of patients with a subgroup of lower and higher frontal alpha power was performed (Fig. 4). We visually scored each patient’s EEG data for the recognition of sedation, and classified patients with a random intercept above the population median alpha power as having a higher alpha power [21]. On the whole, as for patients with low frontal alpha power, the median cumulative time with sedation E-F was lower within the rEEG-guided group compared to that of the routine care group [5 vs. 25, *p* < 0.05], while the median cumulative time with sedation A-B was less in the rEEG-guided group with a higher alpha power (Fig. 5). However, it is noted that no significant difference was found in the POD incidence or any other complications between the two groups (Table 5).

**Table 5:** Subgroup analyses of postoperative complications

	rEEG-guided group	Routine care group	*P*
**POD, no.**			
low frontal alpha power	2(7%)	7(22%)	0.081
high frontal alpha power	0	0	/
**Other systemic complications, no.**			
low frontal alpha power	9(30%)	14(44%)	0.533
high frontal alpha power	10(32%)	15(47%)	0.277

## Discussion

In the randomized controlled trial, the impact of the raw EEG guidance of anesthesia on postoperative complications after surgery in older adults (≥ 60) undergoing major abdominal surgery was assessed. There was no statistically significant difference between the two groups in terms of postoperative respiratory, cardiovascular, neurological and gastrointestinal complications and all-cause mortality at 30 days postoperatively. There was no statistically significant difference in the number of intraoperative BS between the two groups, but rEEG-guided anesthesia management significantly reduced the time patients spent in BS. In post hoc exploratory analyses, low frontal alpha power was found to be independently linked to POD, and that rEEG-guided anesthesia allowed individual regulation of depth of anesthesia to avoid over-anesthesia in patients with a fragile brain, while ensuring the depth of anesthesia required in older patients with a healthy brain.

As the brain is the target organ for general anesthesia, with the continuous research and development of EEG monitoring, some experts advocate the incorporation of EEG-based monitoring into routine anesthetic management. EEG monitoring can avoid too light anesthesia and prevent the occurrence of intraoperative awareness, while EEG monitoring can avoid too deep anesthesia, which usually leads to prolonged recovery time as well as impaired quality of recovery for patients [33]. However, it remains controversial whether EEG monitoring can influence the management of perioperative anesthesia and the extent to which changes in the management of depth of anesthesia can affect the clinical outcomes of patients. In this trial, the absence of an effect of the rEEG guidance on severe systemic complications is consistent with the findings of the existing literature [7, 34, 35]. The majority of large studies have not demonstrated that BIS guidance alters anesthetic administration on average [12]. In study [6] of Short et al., 6500 high-risk older patients in total were randomly divided into the deep (BIS 35) or light (BIS 50) general anesthesia groups, and it was found that the 1-year mortality or any other outcome measure had no difference. In addition, research [7] carried out by Wildes et al. explored the protective influence of BIS monitoring on POD. Despite finding out a connection between a low BIS value and increased risk for mortality, especially if linked to a low anesthetic dose and the “triple low” of hypotension [36], our findings are consistent with more robust randomized trials. The reason for this discrepancy could be that in our study, the blood pressure potential confounder was seen to be mitigated by the anesthetists that selected suitable MAP targets for patients before learning about the treatment allocation. Some randomized controlled trials and meta-analyses have reported that the ability of pEEG-guided anesthetic management to reduce the incidence of POD is largely dependent on the reduction in the use of anesthetic drugs and the duration of EEG suppression [3, 37]. Some studies also suggest that intraoperative BS is associated with the development of POD. In this study, the duration of BS was shorter in the rEEG-guided group than in the routine care group (P < 0.05), and the incidence of POD in patients in the rEEG-guided group was 3% compared with 11% in the routine care group, but the difference between the two groups was not statistically significant, probably due to the small sample size of debilitated patients in this study.

Gamma-aminobutyric acidergic (GABAergic) anesthetics, i.e., sevoflurane, and propofol, generate stereotyped slow (0.1–1 Hz) activity and frontal alpha (8–12 Hz) oscillations of EEG in the process of unconsciousness, both of which are quantified by using a power spectral analysis. A correlation between frontal alpha band activity and preoperative cognitive function has been found which was not present in other EEG bands. Also, EEG alpha band activity strength is linked to age [17], cognitive status [16], antinociception [18], and EEG BS activity [14]. Patients with a lower alpha power are reported to be more prone to the development of BS under anesthesia [32]. Our study shows that a lower frontal alpha power has a closer linkage to delirium risk compared to age, similar to the result that neurophysiologic brain age has a closer connection with delirium compared to chronologic age. Lower intraoperative frontal alpha power may be helpful in identifying patients with poor preoperative cognitive function, leading researchers to propose the concept of the “vulnerable brain” who may experience adverse neurocognitive effects after anesthesia administration. Frontal alpha power under anesthesia is likely to be a marker of “brain age”, and has a closer linkage to POD risk compared to chronologic age, which could be attributed to the biological changes in the brain occurring at variable rates depending on the individual. In a “vulnerable brain” phenotype, it can be found the patient population could obtain the most benefits from a meticulous technique of avoiding excessively deep anesthesia [38]. This study shows that rEEG helps limit unnecessarily excessive anesthetic administration for patients with lower frontal power. What’s more, patients with a healthier brain should be provided with a deeper level of sedation in order to minimize the risk of awareness. In the meantime, significant variation can occur in frontal alpha power, which reflects the neurological status of patients.

This study has several limitations. Firstly, the same anesthesiologist may manage patients in either the rEEG-guided group or the routine care group. Although the anesthesiologists managing the routine care group were masked to the EEG, their previous experience of offering rEEG-guided anesthesia care could have not only enabled them to know more about the anesthesia propofol maintenance but also potentially affected the dosage level. As a result, this research is likely to under-evaluate the actual difference between rEEG-guided and routine care. Secondly, in this research, the explored EEG data comes from the frontal 4-channel pathway, and therefore the analysis only focuses on frontal alpha oscillations in the EEG during unconsciousness, which cannot evaluate other cortical EEG activity. Therefore, high-density EEG research is needed to improve the reliability and utility in the recognition of vulnerable brains and measuring anesthetic depth. Thirdly, a significant correlation was found between the frontal alpha power and POD probability. Taking into account that frontal alpha power is seen as an underlying brain frailty trait, the intervention effect size may be larger when including more cognitively impaired patients, and therefore further validation within bigger research is needed.

## Conclusions

In summary, this is the first randomized control trial that puts emphasis on the elderly population, to point out rEEG-guided anesthesia care that employs EEG trace and spectrogram is feasible and brings about a modest decrease within intraoperative propofol dosage for vulnerable individuals. Due to the existence of the EEG guidance, easy visualization of anesthesia-induced changes on the brain in real-time is permitted, thereby making it possible to decide which individual needs more (or less) anesthetics and accordingly titrate doses. In the findings, the significance of EEG monitoring with the application of the existing American Society of Anesthesiologists (ASA) standard monitors is stressed to offer efficient personalized anesthesia care.

## Data Availability

The datasets used and/or analysed during the current study available from the corresponding author on reasonable request.

## Abbreviations

ASA: American Society of Anesthesiologists
BIS: Bispectral index
BMI: Body mass index
BS: Burst suppression
BSR: Burst suppression ratio
BP: Blood pressure
CI: Confidence interval
EEG: Electroencephalogram
ECG: Electrocardiogram
GABA: Gamma-aminobutyric acid
ICU: Intensive care unit
MAP: Mean arterial pressure
NRS: Numeric rating scales
pEEG: Processed electroencephalogram
PCIA: Patient controlled intravenous analgesia
PACU: Post-anesthesia care unit
POD: Postoperative delirium
PONV: Postoperative nausea and vomiting
rEEG: Raw electroencephalogram
